# The Hepatitis B Virus Pre-Core Protein p22 Activates Wnt Signaling

**DOI:** 10.3390/cancers12061435

**Published:** 2020-05-31

**Authors:** Bang Manh Tran, Dustin James Flanagan, Gregor Ebert, Nadia Warner, Hoanh Tran, Theodora Fifis, Georgios Kastrappis, Christopher Christophi, Marc Pellegrini, Joseph Torresi, Toby James Phesse, Elizabeth Vincan

**Affiliations:** 1 The Peter Doherty Institute for Infection and Immunity, The University of Melbourne, Melbourne 3000, Australia; manht@unimelb.edu.au (B.M.T.); D.Flanagan@beatson.gla.ac.uk (D.J.F.); 2 Cancer Research UK Beatson Institute, Glasgow G61 1BD, UK; 3 The Walter and Eliza Hall Institute of Medical Research, Parkville 3052, Australia; ebert@wehi.edu.au (G.E.); tran.h@wehi.edu.au (H.T.); pellegrini@wehi.edu.au (M.P.); 4 Department of Medical Biology, The University of Melbourne, Melbourne 3010, Australia; 5 Victorian Infectious Diseases Reference Laboratory, The Peter Doherty Institute for Infection and Immunity, Melbourne 3000, Australia; Nadia.Warner@vidrl.org.au; 6 Department of Surgery, Austin Health, The University of Melbourne, Melbourne 3010, Australia; tfifis@unimelb.edu.au (T.F.); g.kastrappis@student.unimelb.edu.au (G.K.); c.christophi@unimelb.edu.au (C.C.); 7 Department of Microbiology and Immunology, The Peter Doherty Institute for Infection and Immunity, The University of Melbourne, Melbourne 3000, Australia; josepht@unimelb.edu.au; 8 European Cancer Stem Cell Research Institute, Cardiff University, Cardiff CF24 4HQ, UK; 9 School of Pharmacy and Biomedical Sciences, Curtin University, Perth, WA 6102, Australia

**Keywords:** Wnt signaling, hepatitis B virus, HBV, cancer, liver cancer, β-catenin, TCF/LEF

## Abstract

An emerging theme for Wnt-addicted cancers is that the pathway is regulated at multiple steps via various mechanisms. Infection with hepatitis B virus (HBV) is a major risk factor for liver cancer, as is deregulated Wnt signaling, however, the interaction between these two causes is poorly understood. To investigate this interaction, we screened the effect of the various HBV proteins for their effect on Wnt/β-catenin signaling and identified the pre-core protein p22 as a novel and potent activator of TCF/β-catenin transcription. The effect of p22 on TCF/β-catenin transcription was dose dependent and inhibited by dominant-negative TCF4. HBV p22 activated synthetic and native Wnt target gene promoter reporters, and TCF/β-catenin target gene expression in vivo. Importantly, HBV p22 activated Wnt signaling on its own and in addition to Wnt or β-catenin induced Wnt signaling. Furthermore, HBV p22 elevated TCF/β-catenin transcription above constitutive activation in colon cancer cells due to mutations in downstream genes of the Wnt pathway, namely *APC* and *CTNNB1*. Collectively, our data identifies a previously unappreciated role for the HBV pre-core protein p22 in elevating Wnt signaling. Understanding the molecular mechanisms of p22 activity will provide insight into how Wnt signaling is fine-tuned in cancer.

## 1. Introduction

Liver cancer is the second most common cause of cancer deaths worldwide and is projected to increase by ~40% by 2030 [1]. The most common type of liver cancer is hepatocellular carcinoma (HCC), which has very limited treatment options and a poor prognosis because it is usually diagnosed at a late stage [2]. The Wnt signal transduction pathway is aberrantly activated in most cases of HCC and mutations to the catenin beta 1 (*CTNNB1*) gene, the gene that codes for β-catenin, occurs in up to 40% of cases making it the most frequent mutation in HCC [3,4]. β-Catenin is the main effector of the canonical Wnt signaling pathway [5] and these mutations to *CTNNB1* lead to constitutive activation of Wnt signaling [6,7]. Liver cancer is also linked to chronic infection with the hepatitis B virus (HBV) that leads to cirrhosis and accounts for 50% of HCC cases [8]. Here, we investigated the oncogenic interplay between these two drivers of liver cancer, namely HBV and Wnt signaling.

Wnt/β-catenin signaling is activated by the coupling of Wnt to its cognate receptor, Frizzled (FZD), which initiates a series of events in the cytoplasm that leads to the activation of (TCF)/lymphoid enhancer factor (LEF)/β-catenin (referred to as TCF/β-catenin for simplicity from here on) mediated gene transcription. In the absence of Wnt, β-catenin is primarily engaged at cell-cell adherens junctions and any free β-catenin is cleared by a cytoplasmic destruction complex that contains several proteins, including Axin, adenomatous polyposis coli (APC), glycogen synthase kinase 3 (GSK3) and casein kinase 1 (CK1) [5]. Free, cytoplasmic β-catenin associates with the destruction complex and is sequentially phosphorylated by CK1 and GSK3 at its N-terminus, a post-translational modification that targets it for ubiquitylation and proteasomal degradation. However, upon activation of Wnt-FZD signaling, GSK3 enzyme activity is inhibited and β-catenin escapes phosphorylation and subsequent degradation, accumulates in the cytoplasm and translocates into the nucleus where it complexes with the enhanceosome to initiate the TCF/β-catenin target gene transcription [9]. In liver cancer, the phosphorylation sites of β-catenin are absent due to mutations to the *CTNNB1* gene, leading to the constitutive activation of Wnt/β-catenin signaling [3,4,10]. 

Another common etiologic factor in liver cancer is HBV infection [10,11]. HBV is an enveloped DNA virus whose genome codes for four overlapping genes, namely the envelope or surface (*S*) gene, the core (*C*) gene, the *X* gene and the polymerase (*P*) gene. The protein products include the surface antigens coded by the *S* gene, the capsid core proteins coded by the *C* gene and the HBx protein coded by the *X* gene. Post-translational processing of the HBV pre-core protein (p25) yields the HBV e antigen (HBeAg, p17) via a p22 intermediate [12]. The HBx protein has been extensively studied for its effects on Wnt/β-catenin signaling [13], however, much less is known about the potential oncogenic interplay with the other HBV proteins. Here, we performed a screen to determine the effects of HBV proteins on Wnt/β-catenin signaling and identified p22, the HBe precursor protein, as a potent activator on its own and in conjunction with active Wnt signaling. Importantly, p22 activated Wnt/β-catenin signaling in colon cancer cells that harbor mutations in intracellular components of the Wnt signaling cascade that result in constitutive activation of signaling. Concomitant regulation of Wnt signaling at multiple levels of the signaling cascade via various mechanisms (genetic, epigenetic, post-translational etc.) to achieve the “just right” level of Wnt signaling for a particular process is a common theme emerging for Wnt-addicted cancers [14,15,16] and here, we demonstrate that HBV p22 might contribute to our understanding of this fine tuning in cancer. 

## 2. Results

### 2.1. Effect of HBV Proteins on TCF-β-Catenin Transcription 

**Figure 1:**
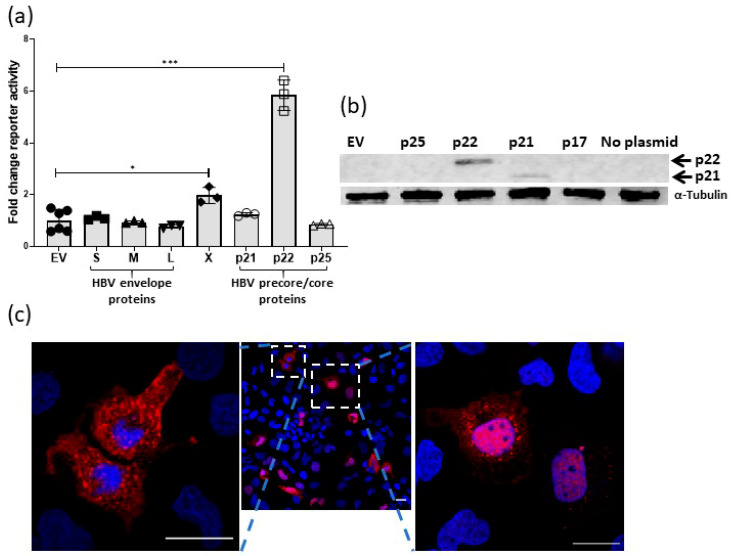
Wnt signaling activation is induced by hepatitis B virus (HBV) precore protein p22. (**a**) Effect of various HBV proteins on TCF/β-catenin transcription activity in Huh7 cells, was determined by reporter activity (sTOPflash reporter) and is shown as fold change relative to empty vector (EV) (mean ± SEM, * *p* < 0.05, *** *p* < 0.0001 Student *t*-test, *n* ≥ 3 independent experiments for each data point) (**b**) Expression of protein from the indicated plasmids transfected in Huh7 cells was confirmed by immunoblot. Lysates prepared from Huh7 cells transfected with EV and the parental, un-transfected cells served as negative controls. Lysate from HBV core p21 transfected Huh7 cells was used as a positive control. The membrane was stained with anti-HBc antibody first, then re-probed with anti α-tubulin antibody. (**c**) Huh7 cells were transfected with p22 plasmid and p22 protein expression (red) and localization detected with anti-HBV core antibody and confocal microscopy (nuclei are blue). Scale bars = 20 µM.

To investigate novel mechanisms of oncogenic interaction between HBV and Wnt signaling we screened the ability of various HBV proteins (Figure S1) for their effect of TCF/β-catenin transcription in the presence of Wnt stimulation (Wnt3a conditioned medium). TCF/β-catenin transcription was detected using the TCF reporter, super TOPflash (sTOPflash), which contains eight TCF response elements upstream of a minimal TK (Thymidine Kinase) promoter and sFOPflash, which has the TCF sites mutated [17,18]. The HBx protein activated TCF/β-catenin transcription above Wnt stimulation, however, the pre-core protein p22 was able to increase Wnt activity to a level markedly greater than the HBx protein (Figure 1a). The HBV envelope proteins did not activate reporter activity, nor did the pre-core precursor p25 or core p21, despite significant overlap in the amino acid sequence between the core/precore proteins (Figure S1). The precore contains the genetic sequence of two different proteins, the core protein HBc (p21) (183 amino acids) and precore polypeptide p25 (212 amino acids). They differ only by 29 amino acids at the N-terminus as p25 retains the signal sequence. The cleavage of 19 amino acids from this signal sequence releases cytosolic p22. P22 is further truncated, losing the arginine-rich C-terminal domain, to yield HBe (p17), which is secreted [19]. Expression of p22 was confirmed by immunoblot on whole cell lysates prepared from transfected Huh7 cells using an anti-HBc antibody and, as shown by others [19], neither p17 nor p25 were detected by immunoblot (Figure 1b and Figure S2). HBV p17 and p25 were detected by confocal immunofluorescence in transfected Huh7 cells (Figure S3). Confocal microscopy of Huh7 cells transfected with pCI-p22 and the same anti-core antibody showed diffuse cytoplasmic, diffuse nuclear and, cytoplasmic puncta (Figure 1c and Figure S3) placing p22 in the cellular compartments where Wnt signaling components are found [20]. 

### 2.2. HBV p22 Activates TCF-β-Catenin Transcription

**Figure 2:**
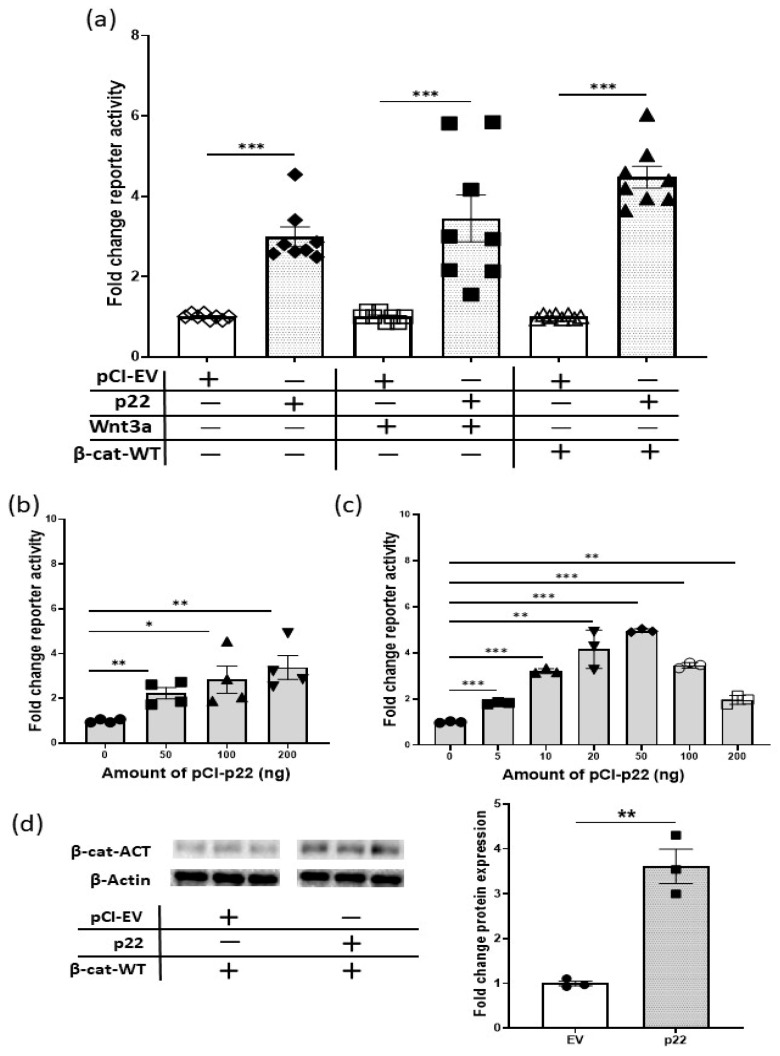
HBV p22 stimulates Wnt signaling in Huh7 cells. (**a**) The effect of HBV p22 alone or in addition to stimulation by Wnt 3a or wildtype β-catenin (β-cat-WT) on TCF/β-catenin transcription in Huh7 cells, was determined by reporter activity (sTOPflash reporter) and is shown as fold change relative to empty vector (EV) (mean ± SEM, *** *p* < 0.0001 Student *t*-test, *n* = 8 independent experiments). (**b**) Huh7 cells were transfected with the indicated amounts of p22 expression plasmid. The figure shows the dose-dependent effect of HBV p22 on TCF/β-catenin transcription activity (sTOPflash reporter) (mean ± SEM, * *p* < 0.05, ** *p* < 0.001 Student *t*-test, *n* = 4 independent experiments). (**c**) Huh7 cells were transfected with the indicated amounts of p22 and 100 ng of wild-type β-catenin expression plasmids. Co-expression of 5–50 ng p22 increased TCF/β-catenin transcription activity (sTOPflash reporter) mediated by wild-type β-catenin; reporter activity decreased when 100 or 200 ng p22 was co-transfected with wild-type β-catenin (mean ± SEM, ** *p* < 0.001, *** *p* < 0.0001 Student *t*-test, *n* = 3 independent experiments). (**d**) Immunoblot analysis for the transcriptionally active form of β-catenin (β-cat-ACT) on lysates prepared from Huh7 cells co-transfected with 100 ng wild-type β-catenin, 100 ng of p22 or equivalent EV expression plasmids. The membrane was stripped and re-probed with anti-actin antibody. The bar graph shows quantitative analysis for the levels of detected active β-catenin using Image Lab software and normalized for β-actin levels (mean ± SEM, ** *p* < 0.001 Student *t*-test, *n* = 3 samples).

Next, we demonstrated that p22 activates Wnt signaling on its own and can increase Wnt signaling activity in cells, which are stimulated with either Wnt3a or ectopic over-expression of full length, wild type β-catenin (β-cat-WT) (Figure 2a). The stimulatory effect of p22 on reporter activity was dose-dependent (Figure 2b) and decreased at the higher levels of p22 in the presence of β-cat-WT (Figure 2c). Notably, the levels of transcriptionally active non-phosphorylated β-catenin (β-cat-ACT) [21,22] were increased above that seen with β-cat-WT when p22 was co-expressed (Figure 2d and Figure S4). In the presence of active Wnt signaling, β-catenin escapes phosphorylation and subsequent degradation, and the elevated levels of β-cat-ACT confirm this mechanism for p22 activation of TCF/β-catenin transcription. Data to illustrate the comparative reporter activity between the different conditions is shown in Figure S5.

During natural HBV infection, p22 is processed to p17 or HBV e antigen (HBeAg) and secreted into the extracellular space [19]. We confirmed that the transfected p22 is processed to p17 by detecting and quantifying HBeAg in the supernatant of transfected Huh7 cells (Figure S6). Notably, ectopically expressed p17 or p25 did not activate sTOPflash reporter activity above activation by β-catenin (Figure S7).

### 2.3. HBV p22 Activates Native TCF/β-Catenin Promoters

**Figure 3:**
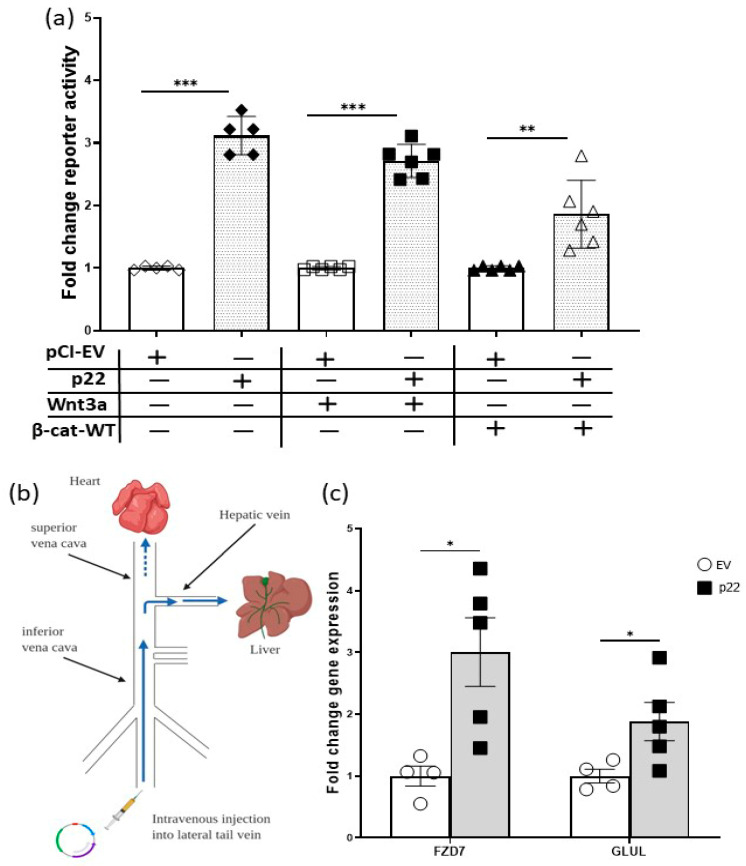
HBV p22 activates TCF/β-target gene native promoters. (**a**) Effect of HBV p22 on FZD7-native promoter reporter activity, with and without stimulation with Wnt3a or 100 ng wild-type β-catenin (β-cat-WT), in Huh7 cells was determined by luciferase activity (pFz7-prom reporter) and is shown as fold change relative to empty vector (EV) (mean ± SEM, ** *p* < 0.001, *** *p* < 0.0001 Student *t*-test, *n* = 6 independent experiments). (**b**) Schematic diagram of hydrodynamic tail-vein injection in mice (adapted from [31]). (**c**) Expression of TCF/β-target genes Fzd7 and glutamine synthase (Glul) was increased in mouse livers 20 days post HDI injection of p22. Gene expression was determined by qRT-PCR and is shown relative to empty vector (EV) (mean ± SEM, * *p* < 0.05 Student *t*-test, *n* ≥ 4 mice).

Next, we tested the ability of p22 to activate native TCF/β-catenin target gene promoters. First, we used our previously characterized Frizzled-7 (FZD7) promoter reporter, pFz7-prom [23]. FZD7 is a TCF/β-catenin target gene [23,24] and forms a positive feedback loop in various cancers, including HCC [25,26,27]. As shown above with the sTOPflash reporter (Figure 2a), HBV p22 activated the pFz7-prom on its own, and in the context of Wnt3a stimulation or β-cat-WT over-expression (Figure 3a).

Secondly, given that Wnt signaling is dependent on a three-dimentional tissue context [28], we tested the ability of p22 to activate native TCF/β-catenin target gene promoters in the liver in vivo. HBV is an exquisitely human hepatotropic virus and does not infect mouse hepatocytes. However, using hydrodynamic tail vein injection (HDI) plasmids can be introduced into mouse hepatocytes in live animals [29]. A large volume of plasmid containing saline was intravenously injected into mice. This volume overwhelms the heart and is shunted into the hepatic vein and the hepatocytes take up the injected solution (Figure 3b). The mice were culled 6 days and 20 days post HDI and their livers processed for mRNA gene expression analyses using quantitative RT-PCR (qRT-PCR). Expression of Wnt target genes (e.g., Fzd7, Glul) and those that are not target genes (e.g., SOCS3) was determined. At 6 days post-HDI, cyclin D2 and SOCS3 were upregulated by p22 (Figure S8a). Cyclin D2 is upregulated upon activation of Wnt signaling via truncating the *APC* gene and regulates proliferation in this setting [30], suggesting it is a Wnt target gene, however this may be indirect. Fzd7, a Wnt target gene [23,24] shows a trend in upregulation in response to p22 at 6 days post HDI, which was significantly different by 20 days post-HDI (Figure 3c and Figure S8), whilst the expression of another TCF/β-catenin target gene glutamine synthetase (Glul, Figure 3c and Figure S8b) was only upregulated by p22 at day 20, suggesting early and late regulation or signaling thresholds. There were trends towards increased expression of other TCF/β-catenin target genes but these changes did not reach significance (full qRT-PCR gene analyses are shown in Figure S8 and primer sequences in Table S1). Collectively, these data show p22 activates natural promoters of TCF/β-catenin target genes in the context of a human liver cancer cell line Huh7 (Figure 3a) and normal liver hepatocytes in vivo (Figure 3c and Figure S8).

### 2.4. HBV p22 Activates TCF/β-Catenin Transcription in Addition to a Mutation to Downstream Wnt Pathway Components

Thus far, we have demonstrated that p22 activates TCF/β-catenin transcription on its own and in the context of Wnt stimulation and β-cat-WT over-expression. This mimics one scenario of additional Wnt signaling in cancer i.e., signaling from the ligand-receptor complex. Next, we investigated p22 activity in other cancer contexts, namely in the context of mutant intracellular components that constitutively activate the Wnt pathway i.e., truncated APC and stabilized, mutant β-catenin. 

**Figure 4:**
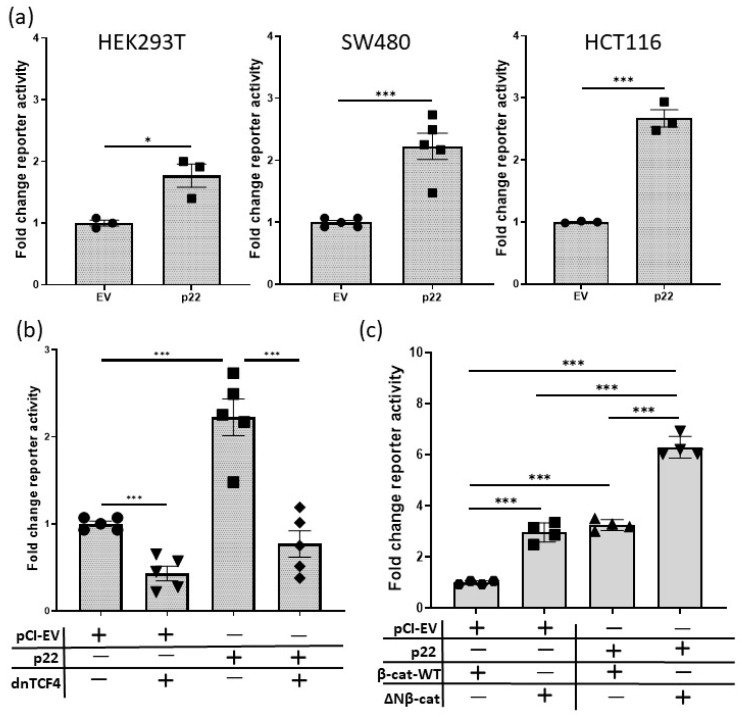
HBV p22 increases TCF/β-catenin signaling in the context of oncogenic activation of the Wnt pathway. (**a**) Effect of 100 ng p22 expression plasmid on TCF/β-catenin transcription activity (sTOPflash reporter) in HEK293T cells with no known mutation or aberrant modulation of Wnt signaling; SW480 cells with truncated, mutant APC, rendering Wnt signaling constitutively active and HCT116 cells with mutation at the N-terminus of β-catenin, making Wnt signaling constitutively active (mean ± SEM, * *p* < 0.05, *** *p* < 0.0001 Student *t*-test, *n* = 3, 5 and 3 experiments, respectively). Reporter activity is expressed relative to empty vector (EV). (**b**) HBV p22 upregulates TCF/β-catenin transcription (sTOPflash reporter) in the context of truncated APC and this upregulation is blocked by dnTCF4. SW480 cells were co-transfected with 100 ng of p22 and dnTCF4 expression plasmids and the reporter activity is expressed relative to EV (mean ± SEM, *** *p* < 0.0001 Student *t*-test, *n* = 5 experiments). (**c**) HBV p22 upregulates TCF/β-catenin transcription (sTOPflash reporter) in the context of mutant, oncogenic β-catenin in Huh7 cells. The effect of co-transfection of 100 ng p22 expression plasmid with 100 ng of wild-type or mutant β-catenin on TCF/β-catenin is shown relative to EV (mean ± SEM, *** *p* < 0.0001 *t*-test, *n* = 4 experiments).

The role of Wnt signaling in cancer has been most extensively studied in colon cancer as Wnt signaling is frequently deregulated in these cancers [32]. Thus, to investigate the effect of p22 in cancer cells with endogenous mutations to intracellular Wnt pathway components, we used colon cancer cell lines SW480 and HCT116 that harbor truncated APC and mutated β-catenin, respectively [18,33]. We also tested the effect of p22 in HEK293T cells that have no known mutations in the Wnt pathway and are known to respond to Wnt [34]. In each cell line (HEK293T, SW480 and HCT116) p22 activated TCF/β-catenin transcription (sTOPflash) above the basal level (Figure 4a). 

There are four mammalian TCF genes and TCF4 is known to be expressed by SW480 cells [18]. Thus, we tested the ability of a dominant negative form of TCF4 (dnTCF4) [18] to inhibit TCF/β-catenin transcription (sTOPflash) in this cell line. As expected, dnTcf4 decreased constitutive Wnt signaling in SW480 cells. HBV p22 increased Wnt signaling in SW480 cells and this increase was reduced by dnTcf4 (Figure 4b). Collectively, these data show p22 regulates Wnt/β-catenin signaling in the context of genetic mutations that initiate Wnt-addicted cancers. 

Next, to further test p22 activity in the context of mutant β-catenin compared to β-cat-WT, we used the N-terminally truncated, oncogenic form of β-catenin (ΔN-β-cat) that lacks the regulatory domains [33]. ΔN-β-Cat increased TCF/β-catenin transcription (sTOPflash) above β-cat-WT to a similar level as p22, while ΔN-β-cat and p22 together elevated reporter activity above either alone (Figure 4c). Data to illustrate comparative reporter activity between some of these different conditions is shown in Figure S5.

## 3. Discussion

The emerging theme for Wnt-addicted cancers is that the pathway is regulated via multiple mechanisms [16]. This has been extensively investigated in colon cancer. Colon cancers frequently harbor truncating mutations to *APC* that yield proteins with impeded function in degrading β-catenin; or oncogenic mutations to the *CTNNB1* gene that remove the destruction complex phosphorylation sites in the N-terminus of β-catenin [35]. The end result of either mutation is the constitutive activation of Wnt signaling and adenoma formation [6,18,33,36,37]. However, Wnt signaling is also deregulated at the level of the ligand/receptor in colon cancer. Naturally occurring inhibitors of Wnt-FZD interaction are silenced by promoter hypermethylation, while Wnts and FZDs are over-expressed (reviewed in [15,25]). Thus, transcription of TCF/β-catenin target genes can be increased or decreased despite a mutation to downstream components of the pathway. Indeed, all Wnt-addicted cancers show concomitant deregulation to Wnt signaling via intracellular and cell surface mechanisms [16]. Consistent with this, a potent anti-tumor effect was demonstrated by blocking FZD7 function in gastric cancer cells with and without mutant *APC* [38].

Notably, liver cancer displays similar Wnt-addicted mechanisms to colon and gastric cancer [16]. Constitutive activation of Wnt signaling in HCC is primarily via mutations to the *CTNNB1* gene that remove the regulatory phosphorylation sites from the N-terminus of β-catenin [3]. However, as in colon and gastric cancer, there is additional regulation of the pathway via over-expression of Wnts and FZDs and epigenetic silencing of naturally occurring inhibitors of Wnt-FZD interaction, for example secreted frizzled related proteins (sFRP) [16,39]. Furthermore, most cases of HCC have a viral etiology and are the culmination of chronic infection with HBV leading to liver disease where HBV proteins, such as HBx, are hypothesized to exert their oncogenic activity, at least in part, through activation of Wnt/β-catenin signaling [8]. Here, we screened the various HBV proteins for their impact on Wnt signaling and demonstrated that another HBV protein, p22, was more potent than HBx. HBV surface proteins (small, middle or large) did not activate TCF/β-catenin transcription. Interestingly, the other pre-core/core proteins (p25, p21 or p17) also did not activate TCF/β-catenin transcription despite significant overlap in their amino acid sequence with p22. Clinical studies show HBe-positivity is a significant independent risk factor of HCC and fatality in chronic HBV-infected patients [40,41]. Furthermore, HBe is produced within the first week after HBV infection in experimental models [42], and thus p22 has the potential to contribute to early events in the transition to cancer. Here, we showed ectopically expressed p22 was localized diffusely in the cytoplasm and nucleus, and in cytoplasmic puncta, indicating potential co-localization with various levels of the Wnt signaling machinery [20]. We also demonstrated Fzd7 and GLUL are induced by p22 in vivo; this shows that genes associate with liver cancer (*Fzd7* [39]) and β-catenin-mediated liver zonation and regeneration (*GLUL,* [43]) are induced by p22 in normal hepatocytes. Furthermore, we demonstrated that p22 can increase TCF/β-catenin transcription on its own and in conjunction with ectopically expressed wild-type or mutant β-catenin; and in colon cancer cells with endogenous mutant *APC* (SW480 cells) or *CTNNB1* (HCT116 cells). Activation of TCF/β-catenin transcription in the SW480 cells by p22 was blocked by dnTCF4, confirming impact specifically on Wnt signaling. 

Collectively, our data identifies HBV p22 as a novel regulator of Wnt signaling in the context of cancer and provides insight into the mechanisms of ‘just right’ Wnt signaling in cancer. Identifying the molecular interactors of p22 will not only be relevant to HCC but to all Wnt-addicted cancers as it is a new tool to investigate context-dependent Wnt signaling. Immunohistochemical studies in colon cancer carcinomas show variable β-catenin staining where β-catenin is primarily membrane-bound in central areas of the tumor, and intense cytoplasmic and nuclear staining in localized regions that are referred to as the invasive front associated with metastasis [44,45]. This implies that Wnt signaling is constrained in cancer cells allowing for bursts of intense signaling for various processes such as metastasis. It remains to be determined if this localized hyperactive Wnt signaling is due to loss of transcriptional repression or activation of transcription. Further investigation of the p22 mechanism of action in ex vivo models systems for example that do not have the limitations of continuous, transformed cell lines and mouse models with respect to human disease [27], might reveal novel avenues of research to help identify new components to selectively harness different aspects of Wnt signaling; for example, blocking oncogenic Wnt signaling while preserving the critical role Wnt signaling provides to ensure the correct regulation of stem cells and homeostasis of many epithelial tissues. Selective regulation of Wnt signaling is at the core of identifying druggable Wnt pathway targets, as the desired outcome for a cancer specific drug that inhibits Wnt is for the drug to allow normal physiological processes to proceed thus reducing the toxicity of a blanket approach of inhibiting Wnt signaling.

## 4. Materials and Methods

### 4.1. Hydrodynamic Injection of Mice 

C57BL/6 mice used in experiments were between 6 and 10 weeks old, and age- and sex-matched (both sexes were used). Hydrodynamic injection (HDI) was performed as we previously described [29]. Briefly, unanesthetized mice were injected intravenously (iv) through the tail vein with 10 μg pCI-p22 or pCI-EV (pCI, Promega, Madison, WI, USA) in a volume of saline equivalent to 8% of the mouse body weight. The injection was performed within 5 s. Mice were killed 6- and 20-days post HDI, their liver resected and processed for analysis. The Walter and Eliza Hall Institute of Medical Research Animal Ethics Committee (AEC) reviewed and approved all animal experiments (AEC number 2017.016).

### 4.2. RNA Extraction and Quantitative RT-PCR (qRT-PCR)

Mouse liver tissues were homogenized in TRizol (Invitrogen, Carlsbad, CA, USA) and total RNA purified, DNAse treated and quantified as previously described [46]. cDNA was synthesized and subjected to qPCR using SYBR green (ABI). Gene expression was calculated relative to the housekeeping gene β2M (2^−ΔΔCt^) as described previously [46] and was expressed as fold change over empty vector (EV).

### 4.3. Cell Lines and Wnt3a Conditioned Medium 

The human cell lines (SW480, HCT116, HEK293T and Huh7) were purchased from ATCC. SW480, HCT116 and HEK293T were maintained in RPMI-1640 supplemented with 20 mM HEPES, 10% (v/v) heat-inactivated fetal bovine serum (FBS), L-glutamine and antibiotics (penicillin and streptomycin). Wnt3a producing L-cells (L-3a) and the parental L-cells (L) were a generous gift from Prof Karl Willert [34]. L-3a, L and the Huh7 cells were maintained in DMEM, 10% (v/v) heat-inactivated FBS, supplemented with L-glutamine and antibiotics. Conditioned medium was prepared from L-3a and L cells in parallel as previously described [34]. 

### 4.4. Transfection and Reporter Assays

Cells were seeded into 24-well plates to reach 60–70% confluence overnight. Cells were transfected with 400 ng total plasmid (empty vector added to keep total plasmid constant) that included 100 ng sTOPflash or sFOPflash (generous gift from Prof Randall T Moon [17]); or 100 ng pGL or pGL-FZD7 promoter [23] and 2 ng *Renilla* luciferase plasmid (phRG-TK, Promega). The pDNA3.1 plasmids expressing β-catenin, ΔNβ-catenin and dnTCF4 were generous gifts from Professor Hans Clevers [18,22] and added at 100 ng/well. The pCI HBV protein expression plasmids were a generous gift from Professor Stephen Locarnini and added at 100 ng, unless indicated otherwise in the text. Cells were transfected using plasmids in OptiMEM (Life Technologies, Grand Island, NY, USA) and Lipofectamine LTX with Plus reagent (Invitrogen) according to the manufacturer’s instructions. Cells were harvested 48 h later and analyzed using the dual luciferase reporter assay system (Promega). For Wnt3a stimulation, cells were treated with 200 µl L-3a or L conditioned medium for 6 h before harvesting in passive lysis buffer. Luciferase activity with control reporters sFOPflash and pGL, and L conditioned medium were negligible. Reporter activity was expressed relative to *Renilla* to the control for transfection efficiency and plotted as fold change over empty vector (EV) as previously described [38].

### 4.5. Immunoblot Analysis

Pre-cast 4–20% polyacrylamide gels (Mini-Protein TGX, Biorad, Hercules, CA, USA were used to separate proteins (Mini-Protein Tetra Cell, Biorad) and transferred onto nitrocellulose membranes using the Transblot-Turbo instrument (Biorad). The membranes were air-dried and blocked overnight in 1% skim milk at 4 °C. The following day, the membranes were incubated in primary antibody for 1 h and bound antibody detected with secondary antibody and ECL (Western Lightening Plus ECL, Perkin Elmer, Waltham, MA, USA). Primary antibody used were mouse anti-HBcAg [C1] (1:1000, Abcam ab8637, Cambridge, UK), mouse anti-αTubulin (1:1000, Abcam ab7291), mouse anti-active β-catenin (1:1000, Merck Millipore 05-665) and mouse anti-β-actin (1:5000, ThermoFisher AM4302, Waltham, MA, USA). Secondary antibody was rabbit anti-mouse polyclonal antibody HRP (1:10,000 Dako P0260, Glostrup, Denmark). 

### 4.6. Immunofluorescenc Confocal Microscopy

Cells were seeded into two-well Nunc Lab-Tek (Thermofisher) chamber slides to reach 60–70% confluence overnight. Cells were transfected with 200 ng plasmid as described above. After 48 h, cells were fixed with 4% paraformaldehyde, permeabilized with 1% Triton-X100 and blocked with 1% FBS and stained with control antibody or anti-HBcAg [C1] (1:400, Abcam ab8637) primary antibody and detected with goat anti-mouse alexa fluor 488 (1:1000 Invitrogen A11029). DAPI (1:2000) was used for nuclear staining and the cells analyzed using Zeiss LSM700 as previously described [38].

### 4.7. Statistical Analysis

The data represent mean ± SEM, where *n* is at least three independent experiments with cell lines or tissue from at least three mice per cohort, unless stated otherwise. The Student *t*-test was used for comparisons and significance was defined as *p* < 0.5.

## 5. Conclusions

Mutations to *APC* and *CTNNB1* are the most frequent mutations in colon and liver cancer, respectively, and are thought to initiate cancer. Here we demonstrate that the HBV precore protein p22 can activate Wnt signaling in these cancer contexts. The ability of p22 to additionally activate Wnt signaling in the context of these mutations indicates oncogenic interplay between HBV infection and Wnt signaling in liver cancer. Furthermore, it is now clear that Wnt-addicted cancers harbor aberrations to Wnt signaling via both intracellular and cell-surface mechanisms [16], thus our findings identify HBV p22 as a novel tool to understand “additional” regulation and “fine-tuning” of Wnt signaling in the context of cancer [14,25]. Understanding the mechanisms that underly normal, wanted Wnt signaling and pathological, unwanted Wnt signaling is an important step for exploiting the Wnt pathway for anti-cancer treatment. 

## Supplementary Materials

The following are available online at https://www.mdpi.com/2072-6694/12/6/1435/s1, Figure S1: Schematic of the HBV genome and the genes encoding various HBV proteins. The HBV genome, depicted as a long purple continuous strand, encodes 7 proteins from 4 open reading frames (ORFs) (surface [S], core [C], polymerase [P], and the X gene [X]), which are shown as large arrows in different colors, and 3 upstream regions [precore (preC), preS1, and preS2]. The transcripts, ORFs, gene regions and protein products are also shown on the right, Figure S2: Expression of protein from the indicated plasmids. Huh7 cells were transfected with the indicated plasmids and protein expression confirmed by immunoblot. Lysates prepared from Huh7 cells transfected with EV and the parental, un-transfected cells served as negative controls. Lysate from HBV core p21 transfected Huh7 cells was used as a positive control. (**a**) The membrane was stained with anti-HBc antibody first, then (**b**) re-probed with anti-tubulin antibody. The boxed areas were used for the cropped blots in Figure 1, Figure S3: Sub-cellular localization of HBV p22. The indicated expression plasmids were transfected into Huh7 and the cells subjected to confocal microscopy following staining with control anti-body and anti-HBV core antibody (red, while DAPI stained nuclei are blue). A higher magnification of the boxed area of the p22 transfected cells is also shown. Scale bars = 20 µM, Figure S4: HBV p22 stimulates Wnt signaling in Huh7 cells. Huh7 cells were co-transfected with 100 ng wild-type β-catenin and the indicated amounts of p22 plasmid and the cell lysates subjected to immunoblot for (**a**) active β-catenin. The membrane was stripped and re-probed with (**b**) anti-actin antibody. The boxed regions in (a) and (b) were used the cropped immunoblots in Figure 2d, Figure S5: Comparative reporter activity in Huh7 cells across the various conditions. The TOPflash and FOPflash reporter activities in Huh7 cells transfected with the indicated plasmids and treated with the indicated conditioned media [L-cell conditioned medium (CM) or L-cell-Wnt3a conditioned medium (Wnt3a CM)] are plotted on the same Y-axis to demonstrate the relative reporter activity between controls [(FOPflash, CM, empty vector (EV)] and test samples (TOPflash, expression plasmids, Wnt3a CM) and are shown as fold change reporter activity relative to FOPflash/EV (Mean ± SEM, Student t test, *n* = 3 experiments). Reporter activity in control samples was negligible, Figure S6: Quantitation of HBeAg levels in the supernatant of transfected Huh7 cells. HBeAg levels in the supernatant fluid of transfected cells were determined (**a**) two days and (**b**) three days after transfection using a commercial Roche anti-HBe kit and Cobas e411 instrument. Cells were transfected with increasing amounts of HBV p22-containing plasmid, from 0 - 200 ng per well, with or without co-transfected 100 ng wild type β-catenin (Mean ± SD, *n* = 3 replicate wells). Transfected p22 was processed to HBeAg and detected in the supernatant, confirming normal processing, Figure S7: Effect HBV p25 and p17 on Wnt signalling. Effect of increasing amounts of transfected HBV precore p17 (**a**) and p25 (**b**) expression plasmids on TCF/β-catenin transcription (sTOPflash reporter) in Huh7 cells co-transfected with 100 ng wild type β-catenin was determined and is shown relative to no p17 and p25, respectively (Mean ± SEM, * *p* < 0.05, Student *t*-test, *n* = 3 experiments), Figure S8: HBV p22 upregulates gene expression in vivo. (**a**) Quantitative RT-PCR analysis of gene expression in livers of mice tail-vein-injected with EV or HBV p22 containing plasmids at 6 days post injection (mean ± SEM, * *p* < 0.05, *n* = 7 and 8 for EV and p22 injected mice, respectively). (**b**) Quantitative RT-PCR analysis of gene expression in livers of mice tail-vein-injected with EV or p22 containing plasmids at 20 days post injection (mean ± SEM, * *p* < 0.05, *n* = 4 and 5 for EV and p22 injected mice, respectively), Table S1: qRT-PCR Primer sequences.
