# Supplementary material for: The Hepatitis B Virus Pre-Core Protein p22 Activates Wnt Signaling
Source: Cancers (Basel). 2020 May 31;12(6):1435. doi: 10.3390/cancers12061435 (PMC7352296; doi:10.3390/cancers12061435)
Supplement: Supplementary file 1 [file cancers-12-01435-s001.pdf]

cancers

MDPI

Supplementary Materials: The hepatitis B virus pre-core protein p22 activates Wnt signaling

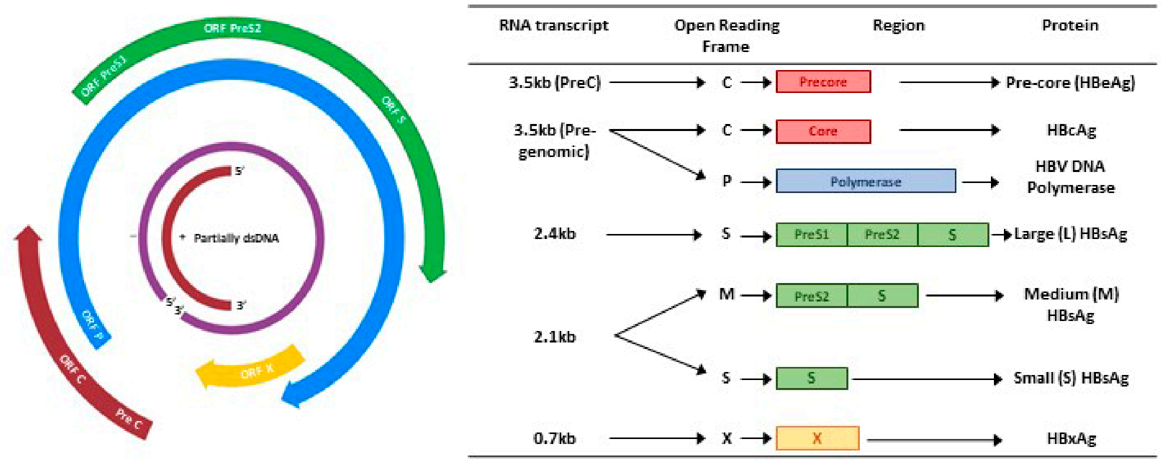

Figure S1. Schematic of the HBV genome and the genes encoding various HBV proteins. The HBV genome, depicted as a long purple continuous strand, encodes 7 proteins from 4 open reading frames (ORFs) (surface [S], core [C], polymerase [P], and the X gene [X]), which are shown as large arrows in different colours, and 3 upstream regions [precore (preC), preS1, and preS2]. The transcripts, ORFs, gene regions and protein products are also shown on the right.

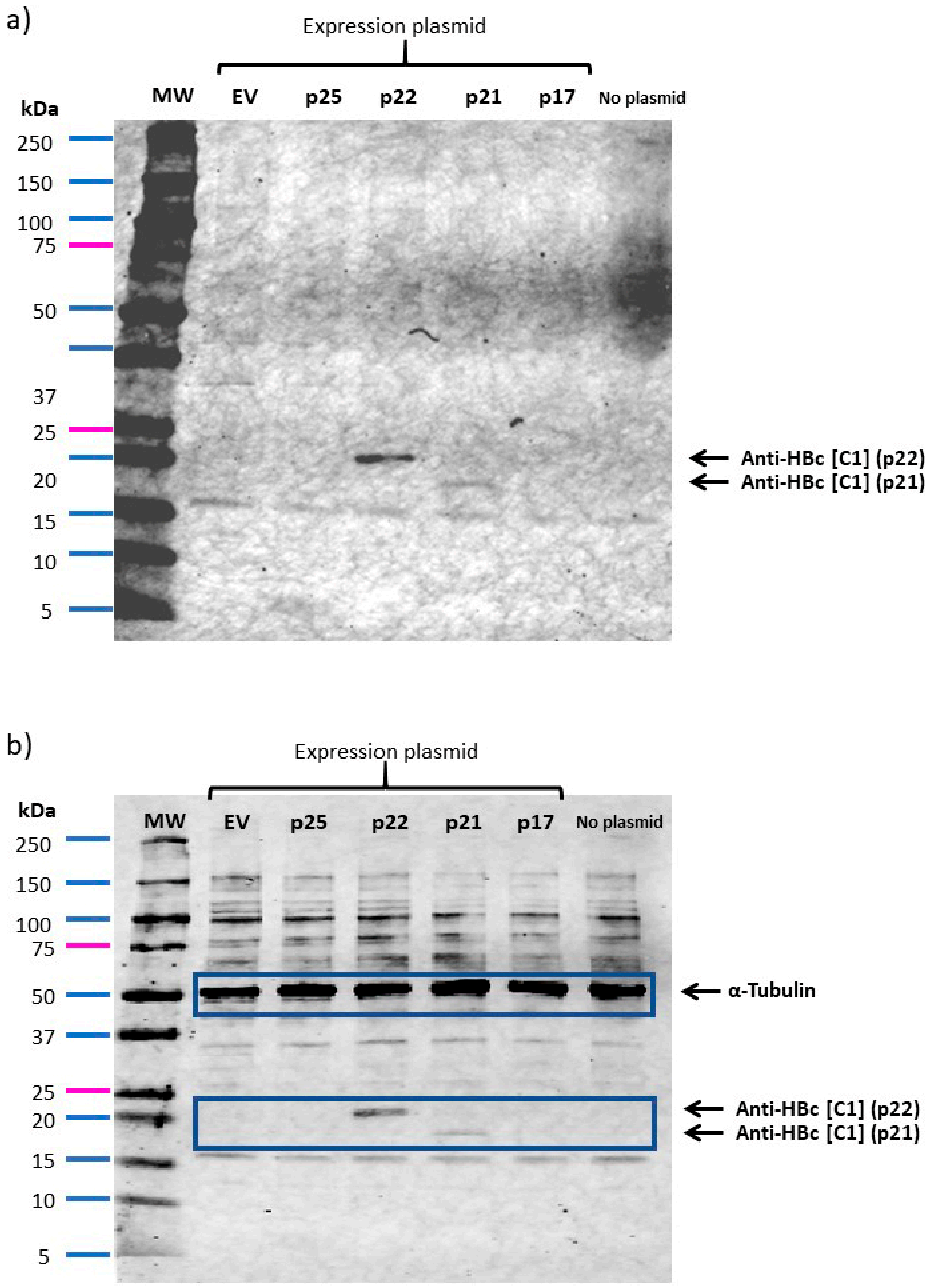

Figure S2. Expression of protein from the indicated plasmids. Huh7 cells were transfected with the indicated plasmids and protein expression confirmed by immunoblot. Lysates prepared from Huh7 cells transfected with EV and the parental, un-transfected cells served as negative controls. Lysate from HBV core p21 transfected Huh7 cells was used as a positive control. (**a**) The membrane was stained with anti-HBc antibody first, then (**b**) re-probed with anti-tubulin antibody. The boxed areas were used for the cropped blots in Figure 1.

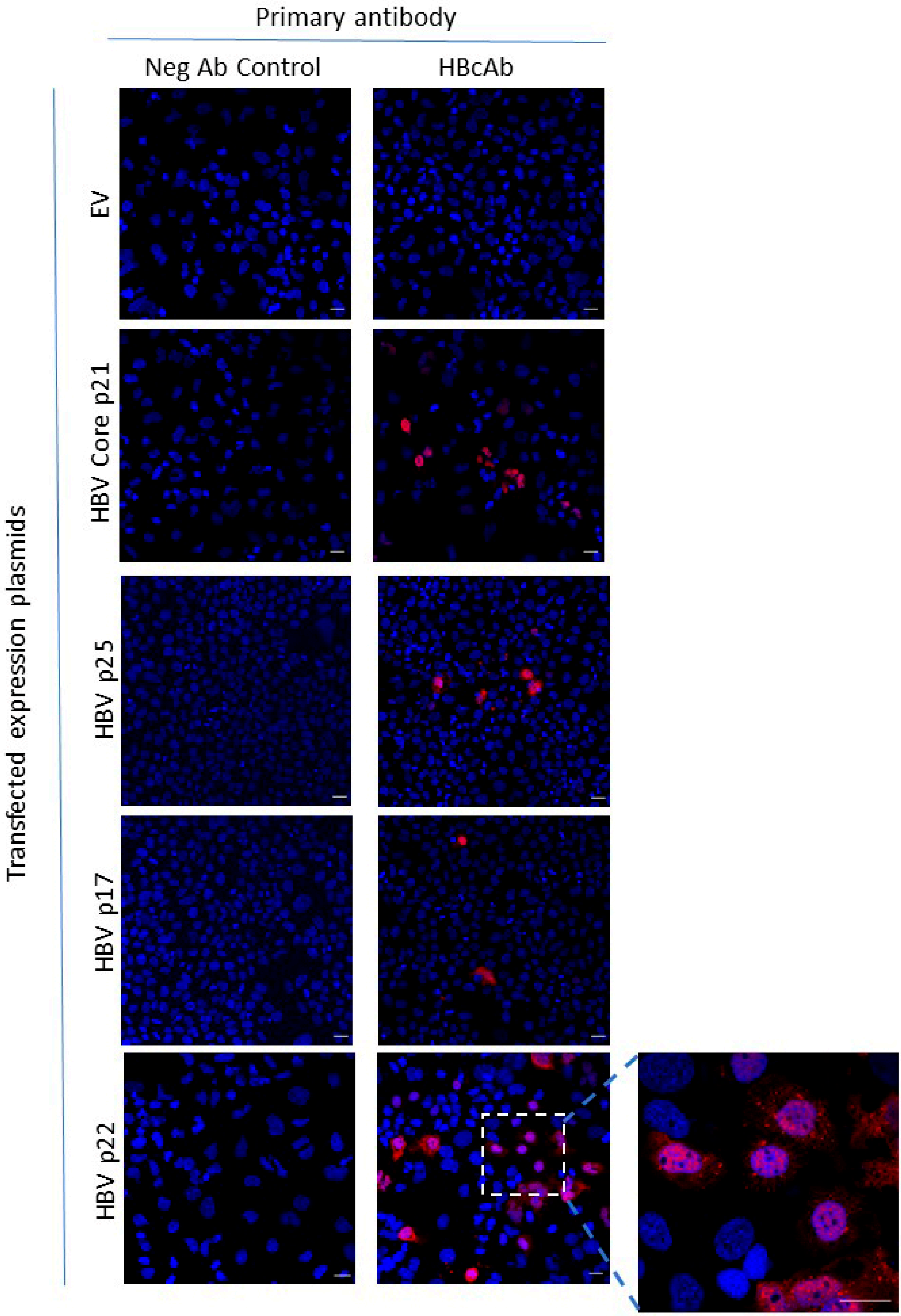

Figure S3. Sub-cellular localization of HBV p22. The indicated expression plasmids were transfected into Huh7 and the cells subjected to confocal microscopy following staining with control anti-body and anti-HBV core antibody (red, while DAPI stained nuclei are blue). A higher magnification of the boxed area of the p22 transfected cells is also shown. Scale bars = 20 μM.

a)

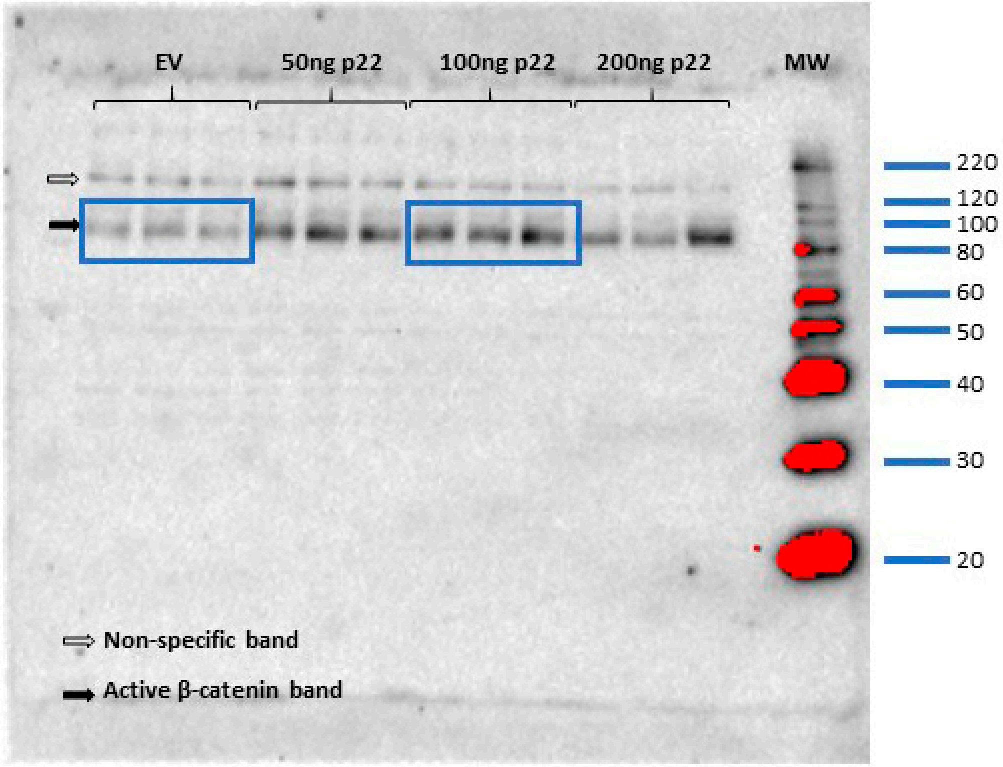

b)

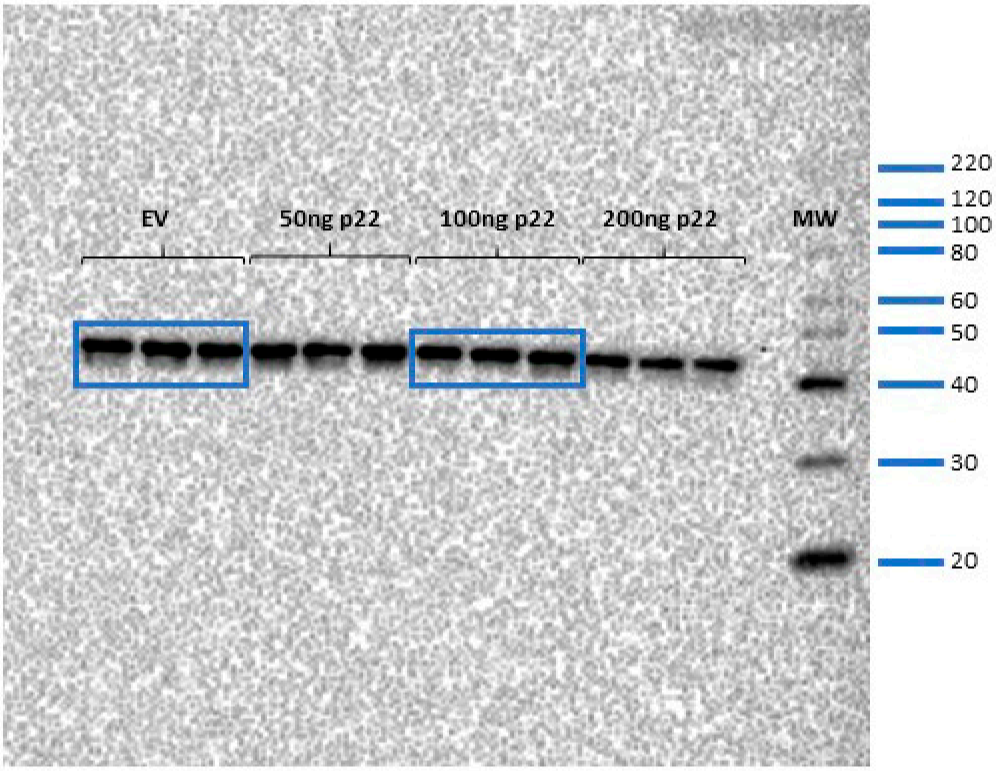

Figure S4. HBV p22 stimulates Wnt signaling in Huh7 cells. Huh7 cells were co-transfected with 100 ng wild-type β-catenin and the indicated amounts of p22 plasmid and the cell lysates subjected to immunoblot for (**a**) active β-catenin. The membrane was stripped and re-probed with (**b**) anti-actin antibody. The boxed regions in (a) and (b) were used the cropped immunoblots in Figure 2d.

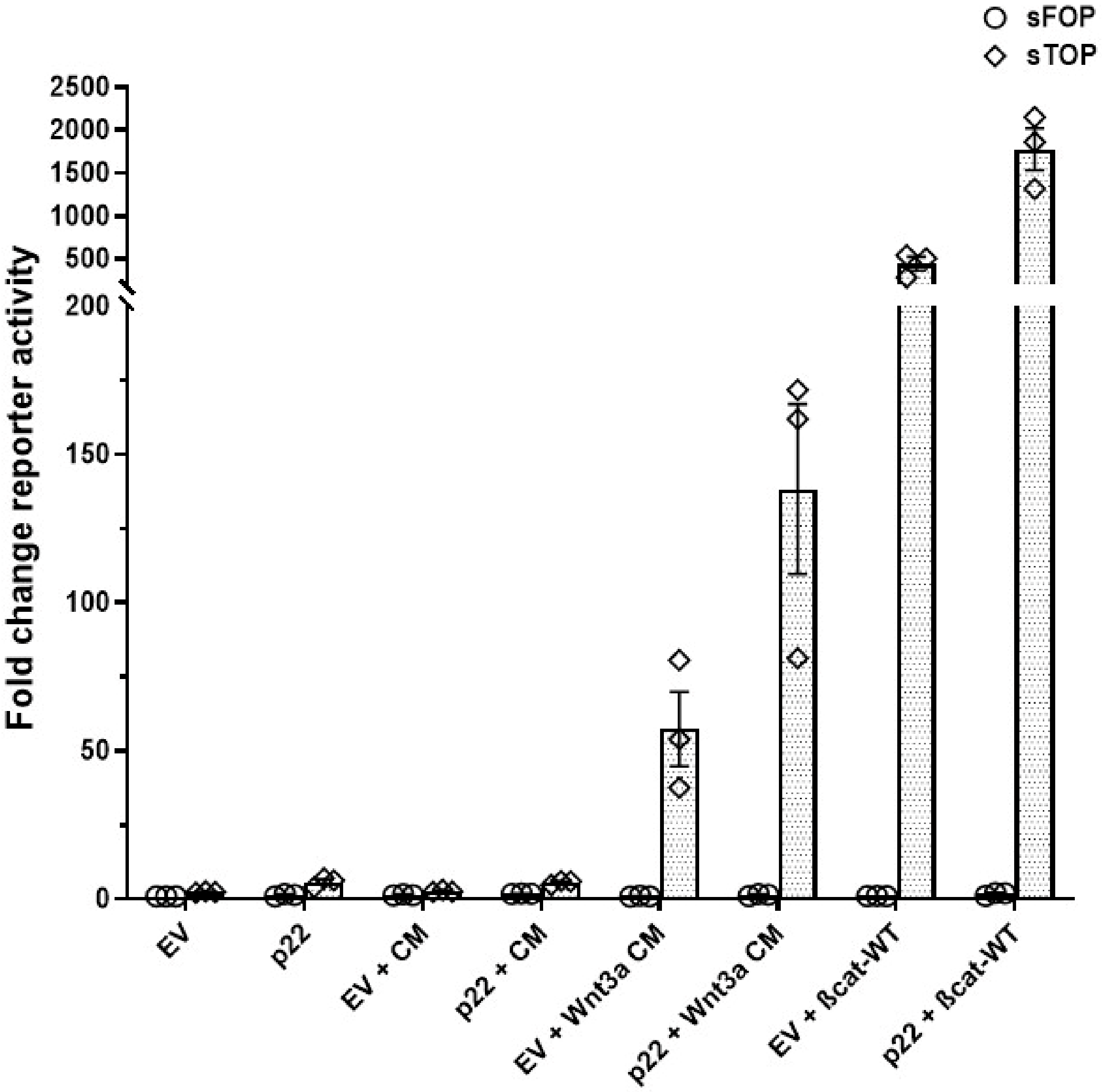

Figure S5. Comparative reporter activity in Huh7 cells across the various conditions. The TOPflash and FOPflash reporter activities in Huh7 cells transfected with the indicated plasmids and treated with the indicated conditioned media [L-cell conditioned medium (CM) or L-cell-Wnt3a conditioned medium (Wnt3a CM)] are plotted on the same Y-axis to demonstrate the relative reporter activity between controls [(FOPflash, CM, empty vector (EV)] and test samples (TOPflash, expression plasmids, Wnt3a CM) and are shown as fold change reporter activity relative to FOPflash/EV (Mean ± SEM, Student t test, n=3 experiments). Reporter activity in control samples was negligible.

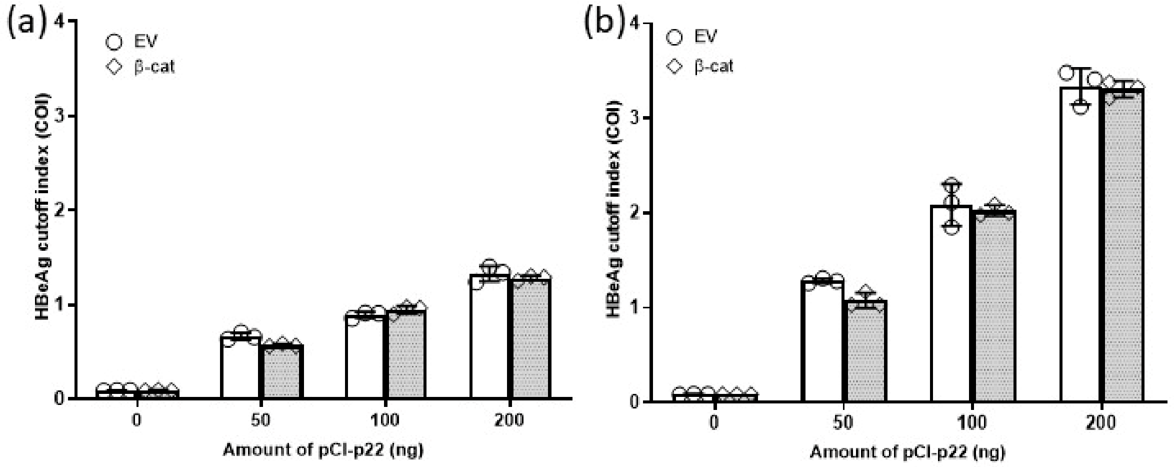

Figure S6. Quantitation of HBeAg levels in the supernatant of transfected Huh7 cells. HBeAg levels in the supernatant fluid of transfected cells were determined (**a**) two days and (**b**) three days after transfection using a commercial Roche anti-HBe kit and Cobas e411 instrument. Cells were transfected with increasing amounts of HBV p22-containing plasmid, from 0 - 200 ng per well, with or without co-transfected 100 ng wild type β-catenin (Mean ± SD, n=3 replicate wells). Transfected p22 was processed to HBeAg and detected in the supernatant, confirming normal processing.

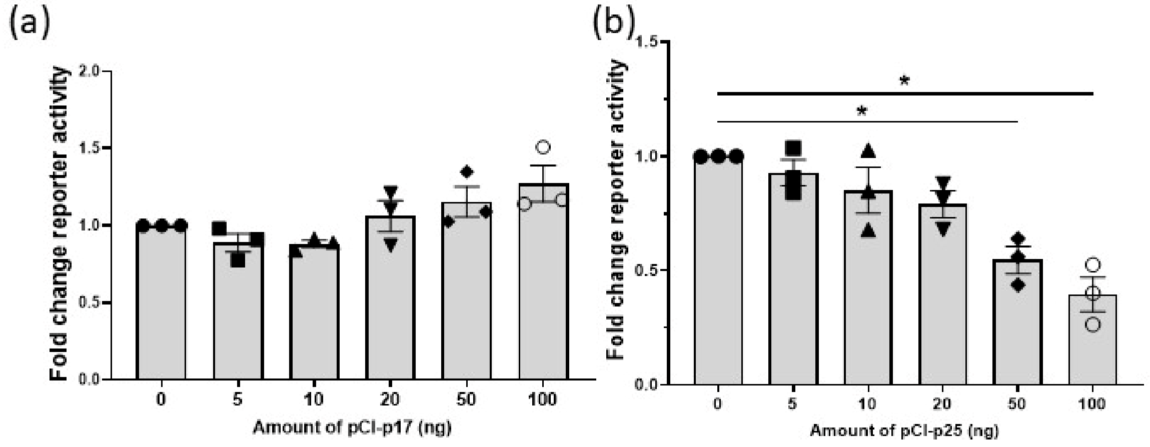

Figure S7. Effect HBV p25 and p17 on Wnt signalling. Effect of increasing amounts of transfected HBV precore p17 (**a**) and p25 (**b**) expression plasmids on TCF/β-catenin transcription (sTOPflash reporter) in Huh7 cells co-transfected with 100 ng wild type β-catenin was determined and is shown relative to no p17 and p25, respectively (Mean ± SEM, *$p<0.05$, Student t-test, n=3 experiments)

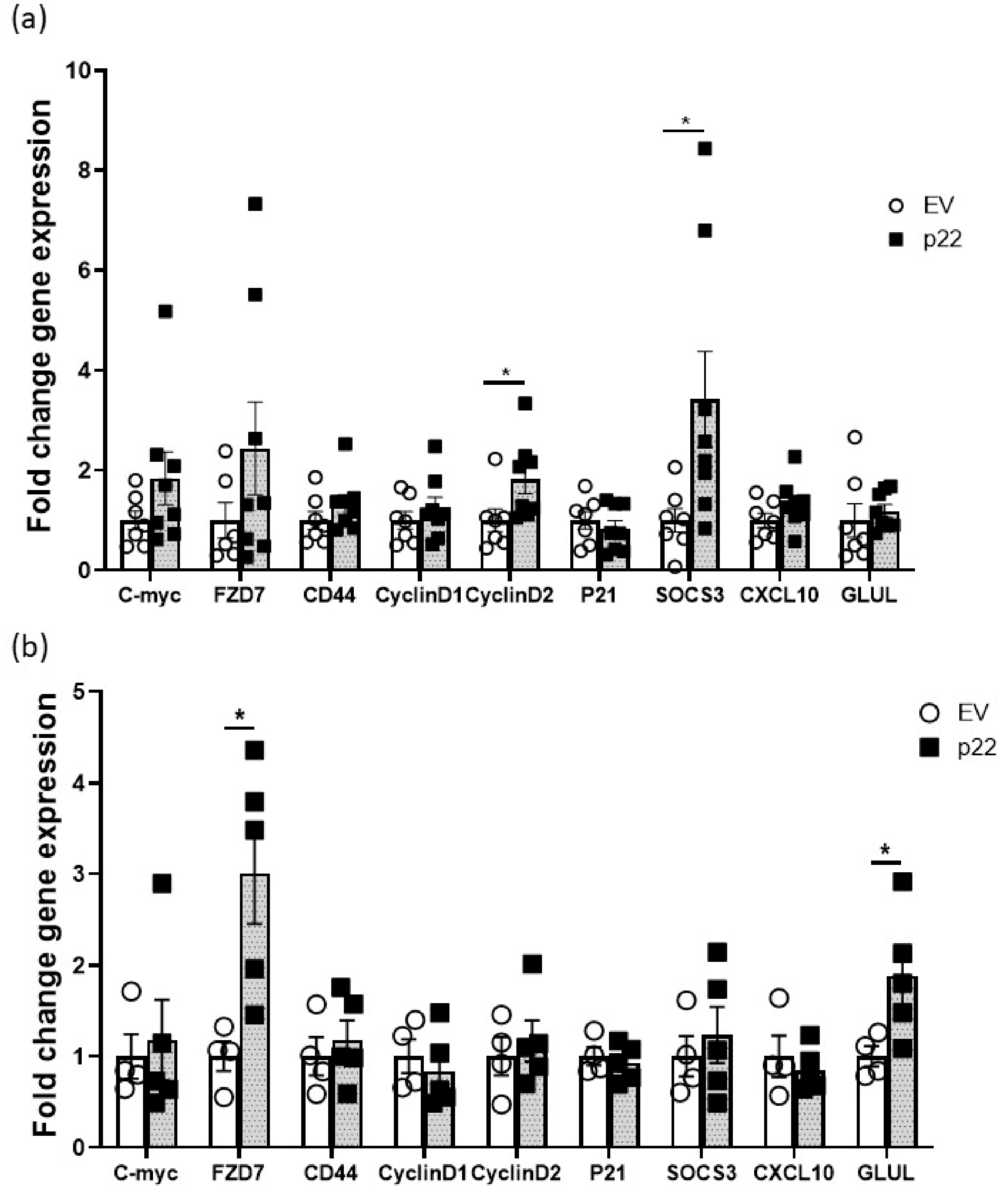

Figure S8. HBV p22 upregulates gene expression *in vivo*. (**a**) Quantitative RT-PCR analysis of gene expression in livers of mice tail-vein-injected with EV or HBV p22 containing plasmids at 6 days post injection (mean ± SEM, *$p<0.05$, n=7 and 8 for EV and p22 injected mice, respectively). (**b**) Quantitative RT-PCR analysis of gene expression in livers of mice tail-vein-injected with EV or p22 containing plasmids at 20 days post injection (mean ± SEM, *$p<0.05$, n=4 and 5 for EV and p22 injected mice, respectively).

Table S1. qRT-PCR Primer sequences

Gene name	Forward Primer	Reverse Primer
C-myc	5'- TAGTGCTGCATGAGGAGACA -3'	5'-GGTTTGCCTCCTCTCCACAG-3'
Fzd7	5'-GCTTCCTAGGTGAGCGTGAC-3'	5'-AACCCGACAGGAAGATGATG-3'
CD44	5'-GTCTGCATCGCGGTCAATAG-3'	5'-GGTCTCTGATGGTTCCTTGTT-3'
CyclinD1	5'-TCGTGGCCTCTAAGATGAAGGA-3'	5'-TCGGGCCGGATAGAGTTG T-3'
CyclinD2	5'-CCTGGATGCTAGAGGTCTGTG-3'	5'-ACCCAGGAGCTGAAGATGG-3'
P21	5'-GCCTTAGCCCTCACTCTGTG-3'	5'-AGCTGGCCTTAGAGGTGACA-3'
SOCS3	5'-GCCACCTACTGAACCCTCCT-3'	5'-ACGGTCTTCCGACAGAGATG-3'
CXCL10	5'-GCTTCCCTATGGCCCTCATT-3'	5'-GACGGTCCGCTGCAACTG-3'
GLUL	5'-TTTATCTTGCATCGGGTGTG-3'	5'- TTGATGTTGGAGGTTTCGTG-3'

© 2020 by the authors. Licensee MDPI, Basel, Switzerland. This article is an open access article distributed under the terms and conditions of the Creative Commons Attribution (CC BY) license (http://creativecommons.org/licenses/by/4.0/).
